# Review on Multispectral Photoacoustic Imaging Using Stimulated Raman Scattering Light Sources

**DOI:** 10.3390/s25113325

**Published:** 2025-05-25

**Authors:** Yuon Song, Sang Min Park, Yongjae Jeong, Jeesu Kim, Hwidon Lee

**Affiliations:** 1 Departments of Cogno-Mechatronics Engineering and Optics & Mechatronics Engineering, Pusan National University, Busan 46241, Republic of Korea; yuonsong@pusan.ac.kr (Y.S.); zxc2726655@pusan.ac.kr (Y.J.); jeesukim@pusan.ac.kr (J.K.); 2 Engineering Research Center for Color-Modulated Extra-Sensory Perception Technology, Pusan National University, Busan 46241, Republic of Korea; psm159@pusan.ac.kr (S.M.P.)

**Keywords:** photoacoustic imaging, stimulated Raman scattering, multispectral imaging, biomedical imaging, light source

## Abstract

Photoacoustic imaging is an advanced biomedical imaging technique that has been widely developed and applied in diverse biomedical studies. By generating optical-absorption-based signals with ultrasound resolution, it enables in vivo visualization of molecular functional information in biological tissues. Extensive research has been conducted to develop the multispectral light sources required for functional photoacoustic imaging. Among the various approaches, multispectral light sources generated using stimulated Raman scattering have shown considerable promise, particularly in photoacoustic microscopy, where achieving multispectral illumination remains challenging. This review summarizes photoacoustic imaging systems that employ stimulated Raman scattering for multispectral light sources and delves into their configurations and applications in the functional analyses of biological tissues. In addition, the review discusses the future directions of multispectral light sources by comparing different technologies based on key factors such as wavelength tunability, repetition rate, and power, which critically affect the accuracy and quality of multispectral photoacoustic imaging.

## 1. Introduction

Photoacoustic imaging (PAI) is an advanced imaging technique that provides the molecular function information of biological tissues by analyzing their optical absorption characteristics [1,2,3]. This technique is based on the photoacoustic (PA) effect in which ultrasound (US) waves (also referred to as PA waves) are generated via the absorption of light and subsequent heat release. PAI enables extracting optical absorption information with relatively high resolution in deep tissues by achieving signals through acoustic wave propagation [4]. This approach offers distinct advantages in biomedical imaging, particularly in deep tissue imaging, compared to that using pure optical imaging techniques, which are often limited by intense light scattering in biological tissues [5]. In addition, unlike conventional US imaging, which detects US waves reflected by changes in acoustic impedance, PAI differentiates various molecular compositions based on the multispectral optical absorption properties of tissues [6].

Similar to other optical imaging modalities, PAI can visualize molecular compositions by analyzing the multispectral responses of biological tissues [7]. Endogenous chromophores such as oxy-hemoglobin (HbO), deoxy-hemoglobin (HbR), melanin, and lipids enable label-free PAI, providing functional information from biological tissues in vivo [8]. Recent advances in PAI systems have extended their application to human tissues [9].

Imaging by incorporating clinically relevant configurations such as free-hand scanning with handheld probes [10,11,12], mobile design [13,14,15], and real-time image generation [16,17,18] has been employed for assessing hemoglobin oxygen saturation (sO_2_) levels to diagnose diseases related to thyroid [19,20,21], breast [22], prostate [23], and skin [24] cancers. Further, various types of exogenous agents have been used for contrast-enhanced PAI [25,26,27]. Applications of optically absorbing nanoparticles functionalized for targeting specific diseases or releasing drugs have been demonstrated in preclinical small animal studies, including in contrast-enhanced imaging [28,29,30], drug delivery monitoring [31], and image-guided therapy [32].

PAI systems can be implemented in three primary configurations: photoacoustic computed tomography (PACT), acoustic-resolution photoacoustic microscopy (AR-PAM), and optical-resolution photoacoustic microscopy (OR-PAM) [33]. PACT utilizes low-frequency US array transducers for imaging wide and deep structures, which produces real-time images of deep tissues within a few centimeters [34,35,36]. AR- and OR-PAM utilize high-frequency single-element transducers for high-resolution imaging, which is often required in small animal studies [37,38,39]. AR-PAM achieves the desired spatial resolution using a focused US transducer (UST), which provides high-resolution images at moderate depths (typically up to 1–2 cm) [40,41,42]. In contrast, OR-PAM offers excellent spatial resolution at shallow depths using tightly focused laser illumination [43,44,45]. All of these configurations have been widely investigated based on the imaging targets. Among them, OR-PAM is found to be particularly effective for high-resolution imaging of superficial tissues such as the eyes [46], ears [47], and skin [48] of small animals.

Generating suitable light sources is a key challenge in implementing multispectral OR-PAM. Although multiple single-wavelength light sources can be used for multispectral imaging [49,50], this approach increases the complexity and overall cost of the system. Multispectral OR-PAM requires light sources with a high pulse repetition rate (PRR), multiwavelength capability, and sufficient pulse energy. Although optical parametric oscillator (OPO) light sources, which are commonly used in this context, provide a wide wavelength range and high pulse energy, they suffer from low PRR (10 Hz to 10 kHz), limiting imaging speed [51,52,53,54]. Ti:sapphire lasers have also been used to provide multiwavelength output with high pulse energy and high PRR [55,56,57]. However, their fixed tunable range of 650–1100 nm, determined by the gain bandwidth of Ti^3+^, may limit their applicability in certain imaging applications. In contrast, supercontinuum light sources feature a broad wavelength range and high PRR. However, their low pulse energy density renders them less effective for accurate multispectral PAI [58,59,60].

Recent advancements have introduced stimulated Raman scattering (SRS) light sources, which demonstrate high PRR, multiwavelength selectivity, narrow linewidth, and pulse energy exceeding several hundred nanojoules. This approach utilizes a nonlinear optical phenomenon in optical fibers, wherein silica molecules within the optical fiber absorb some of the light energy and vibrate, thereby emitting light at shifted wavelengths [61]. The desired wavelengths can be selected for PAI by using proper optical filters [62]. In addition, OR-PAM requires tightly focused light to achieve a high spatial resolution, which necessitates precise laser beam control. Optical fibers offer flexibility and spatial efficiency for light delivery [63,64].

In this review, we discuss the underlying principles and explore recent progress development of multispectral PAM systems incorporating SRS light sources. We begin by discussing the fundamental principles of PAI and SRS generation, and then examine representative multispectral PAM systems, focusing on the wavelength ranges provided by the SRS light sources. Further, we review system configurations and functional imaging capabilities and offer insights into the development of advanced multispectral PAM systems utilizing SRS light sources.

## 2. Principles

### 2.1. Principles of Photoacoustic Imaging

**Figure 1:**
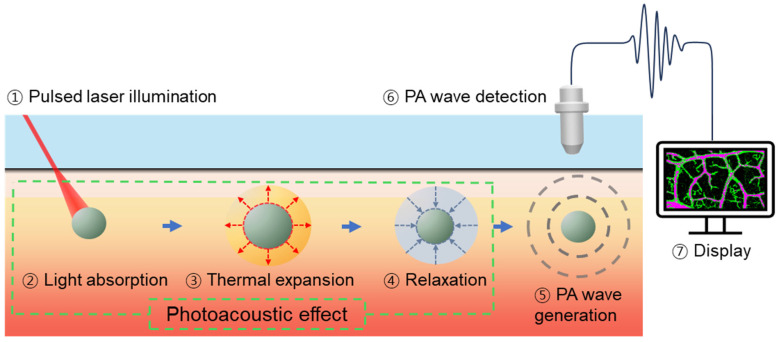
PAI involves the generation and acquisition of PA signals. PAI, photoacoustic imaging; PA, photoacoustic.

PAI involves a series of processes that acquire PA signals in the form of US waves generated by optical absorption and the subsequent heat release in a sample (Figure 1) [65]. The absorber absorbs the energy when a short pulse of light (typically in the nanosecond range) irradiates a region of interest (ROI), resulting in a slight increase in temperature. This small increase in temperature, usually on the order of a few millikelvin, induces thermoelastic expansion in the absorber. However, the material rapidly returns to thermal equilibrium and contracts because the pulse duration is shorter than the time required for the heat energy to dissipate through thermal conduction [1]. Broadband low-amplitude PA waves are generated throughout this process and propagate omnidirectionally. Compared to electromagnetic waves, PA waves have a considerably lower frequency range, which results in reduced scattering and deeper penetration properties, enabling them to reach the surface with minimal attenuation [66]. These waves can be detected and converted into electrical signals using conventional UST, and the signal can be acquired in the time domain based on the distance from the absorbers. Subsequently, the acquired signal is converted into digital data, processed, and reconstructed into an image. The amplitude of the PA signals varies based on several factors, including penetration depth, tissue acoustic properties, and the characteristics of the UST. The most direct cause of PA wave generation is defined as

(1) $$P\propto \Gamma \left(T\right)\cdot \sigma \cdot \mu_{a}\cdot F.$$

Variables that affect the initial pressure P of the PA wave include Γ(T), σ, $\mu_{a}$, and F, which represent the Grüneisen parameter at the local temperature, thermal conversion efficiency, optical absorption coefficient, and optical fluence, respectively.

**Figure 2:**
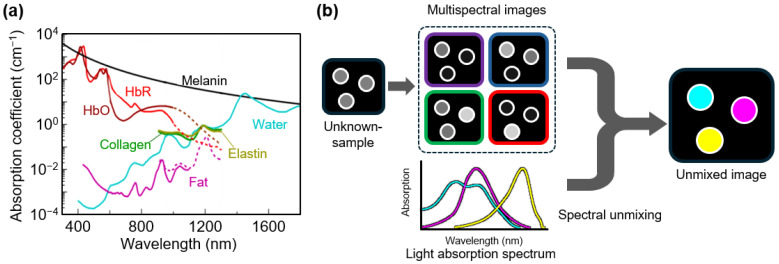
(**a**) Optical absorption spectra of common chromophores in vivo. The dashed lines in HbO and HbR are values at wavelengths greater than 1000 nm. In fat, the solid line is the value from purified pig and the dashed line is the value from human fat. (**b**) Schematic of the spectral unmixing technique using multispectral images and wavelength-specific optical absorption. The outlined colors in the multispectral image indicates the multispectral wavelengths for spectral unmixing. HbO, oxy-hemoglobin; HbR, deoxy-hemoglobin. The images are reproduced with permission from Ref. [67].

Most parameters remain largely unchanged in a fixed image acquisition environment, but the thermal conversion efficiency and optical absorption coefficient are intrinsic properties of the absorber (Figure 2a). In other words, the wavelength at which the maximum PA signal intensity was observed varies depending on the material. The optical absorption coefficient changes with the wavelength, even for the same material, and each substance has a unique nonlinear absorption spectrum [67]. These optical characteristics suggest the potential of functional imaging using multispectral PAI, which offers more than simple structural imaging. A simple comparison of the PA signal intensities at different wavelengths can distinguish specific materials when two or more wavelengths are used, and the proportions of the materials can also be determined by employing additional mathematical models.

Linear spectral unmixing is among the simplest and most effective methods for determining the proportions of different substances when their absorption spectra at various wavelengths are known (Figure 2b) [68]. When the PA signal intensity measured at each pixel is a mixture of multiple components, the contribution of each component can be estimated using a mathematical model based on the following equation.

(2) $$I_{\lambda_{j}}^{P_{i}}=\sum_{k=1}^{m}\;\mu_{\lambda_{j}}^{M_{k}}\cdot C_{M_{k}}^{P_{i}}.$$

At point $P_{i}$ in the image, the intensity I for a specific wavelength $\lambda_{j}$ represents the sum of the product of the concentration C and optical absorption μ at wavelength$\;\lambda_{j}$, which are related to $M_{k}$. For the total pixel *i* of the image, this expression can be expanded to a matrix as

(3) $$\left[\begin{array}{cccc} I_{\lambda_{1}}^{P_{1}} & I_{\lambda_{1}}^{P_{2}} & \ldots & I_{\lambda_{1}}^{P_{i}}\\ I_{\lambda_{2}}^{P_{1}} & I_{\lambda_{2}}^{P_{2}} & \ldots & I_{\lambda_{2}}^{P_{i}}\\ \vdots & \vdots & \ddots & \vdots \\ I_{\lambda_{j}}^{M_{1}} & I_{\lambda_{j}}^{M_{2}} & \ldots & I_{\lambda_{j}}^{P_{i}} \end{array}\right]=\left[\begin{array}{cccc} \mu_{\lambda_{1}}^{M_{1}} & \mu_{\lambda_{1}}^{M_{2}} & \ldots & \mu_{\lambda_{1}}^{M_{k}}\\ \mu_{\lambda_{2}}^{M_{1}} & \mu_{\lambda_{2}}^{M_{2}} & \ldots & \mu_{\lambda_{2}}^{M_{k}}\\ \vdots & \vdots & \ddots & \vdots \\ \mu_{\lambda_{k}}^{M_{1}} & \mu_{\lambda_{k}}^{M_{2}} & \ldots & \mu_{\lambda_{k}}^{M_{k}} \end{array}\right]\times \left[\begin{array}{cccc} C_{M_{1}}^{P_{1}} & C_{M_{1}}^{P_{2}} & \ldots & C_{M_{1}}^{P_{i}}\\ C_{M_{2}}^{P_{1}} & C_{M_{2}}^{P_{2}} & \ldots & C_{M_{2}}^{P_{i}}\\ \vdots & \vdots & \ddots & \vdots \\ C_{M_{k}}^{P_{1}} & C_{M_{k}}^{P_{2}} & \ldots & C_{M_{k}}^{P_{i}} \end{array}\right].$$

Alternatively, this expression can be expressed in matrix notation as

(4) I=M×C ,

where intensity I and optical absorption coefficient M are known data. The equation can be transformed to solve for concentration C using the Moore–Penrose pseudoinverse operation [69].

(5) $$C={\left(M^{T}M\right)}^{-1}\cdot M^{T}I.$$

Recently, blind source separation algorithms [70] and nonlinear spectral unmixing techniques using deep learning [71] have been proposed to compensate for nonlinear relationships attributed to optical attenuation and US scattering in tissues. Given the advancements in these techniques, multispectral PAI can provide more reliable results. Through this series of processes, multispectral PAI can be used to quantitatively determine the proportion of each chromophore in the ROI, which makes it useful for quantitatively monitoring substances and identifying the causes or effects of physiological responses in vivo.

### 2.2. Principles of Stimulated Raman Scattering

**Figure 3:**
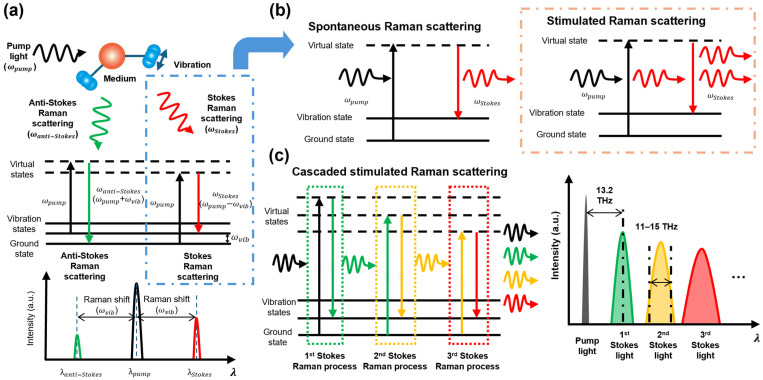
(**a**) Schematic of Raman scattering mechanism: Stokes and anti-Stokes Raman scattering. (**b**) Comparison between spontaneous Raman scattering and SRS. (**c**) Illustration of cascaded SRS, which shows the sequential generation of higher-order Stokes light and corresponding spectrum. SRS, stimulated Raman scattering; $\omega_{pump}$, pump light frequency; $\omega_{Stokes}$, Stokes Raman frequency; $\omega_{anti-Stokes}$, anti-Stokes Raman frequency; $\omega_{vib}$, vibration frequency; $\lambda_{pump}$, pump wavelength; $\lambda_{Stokes}$, Stokes wavelength; $\lambda_{anti-Stokes}$, anti-Stokes wavelength.

Raman scattering is a nonlinear optical phenomenon that has become a crucial technique for analyzing the vibrational properties of substances, including biological samples, polymers, nanomaterials, and various chemical compounds. The photon is excited to higher virtual states when incident light interacts with the target molecules, and then, it generates scattered light by releasing energy through photon relaxation (Figure 3a). The energy of the resulting scattered light is determined by the vibrational modes of the chemical bonds within the target molecules. Most photons are scattered elastically, producing scattered light with retained energy comparable to that of the pump light. This process is referred to as Rayleigh scattering. In contrast, the energy of the scattered light is either higher or lower than that of the pump light when the pump light is scattered inelastically, which is referred to as Raman scattering.

Raman scattering can be categorized as Stokes and anti-Stokes Raman scattering. This can be understood as the interaction between the pump light and vibrational states of the molecules. Raman scattering is considered temperature-dependent because this interaction depends on the vibrational energy levels of the molecules. In Stokes Raman scattering, the excited photon is relaxed into the vibrational states, releasing lower (i.e., longer-wavelength) light energy than that of the pump light. In contrast, anti-Stokes Raman scattering starts from the vibrational state and releases higher-wavelength (i.e., shorter-wavelength) light. At room temperature, Stokes Raman scattering is dominant over anti-Stokes Raman scattering because most molecules are relaxed in their ground state. Consequently, most Raman-scattering applications focus on Stokes Raman scattering, which generates scattered light with longer wavelengths.

Raman scattering can be further classified into spontaneous Raman scattering and SRS (Figure 3b). In spontaneous Raman scattering, the Stokes light is generated through spontaneous emission after excitation to a virtual state using a pump light, and this results in incoherent light. In contrast, SRS occurs when both the pump light and Stokes light interact with the molecules, thereby inducing a stimulated emission that produces coherent Stokes light. SRS generates highly directional Stokes light, which typically has a higher magnitude than that generated using spontaneous Raman scattering. When the peak power of the pump light is sufficiently high, the generated Stokes light interacts with the molecules and generates higher-order Stokes light (Figure 3c). This process, referred to as cascaded SRS, can be continued for the sequential generation of higher-order Stokes light.

While Raman crystals can also be used to generate Raman gain, optical fibers can be considered a suitable medium for cascaded SRS because of the low attenuation of photons, which can increase the interaction length for SRS. Depending on the crystal medium, the Stokes wavelength can be tuned, and high peak power output can be achieved in Raman crystal [72]. However, crystal-based SRS sources generally support a limited number of Stokes orders compared to fiber-based SRS, which offer more flexibility for generating multiple wavelengths through cascaded processes. In addition, optical fibers can transmit high-power light through core diameters smaller than 10 μm, which can significantly enhance the nonlinearity of the medium [73,74]. The generated Stokes light is rapidly amplified by the SRS when a pump light enters the optical fiber. Consequently, the cascaded SRS process is efficiently initiated. The threshold peak power ($P_{th}$) of the pump laser required to initiate each Stokes Raman process in cascaded SRS can be expressed as follows [75]:

(6) $$P_{th}\approx \frac{16\cdot A_{eff}}{k_{p}\cdot L_{eff}\;\cdot \;g_{R}}\;,$$

where $g_{R}$ denotes the Raman gain coefficient of the medium. The polarization factor ($0.5\leq k_{p}\leq 1$) increases when the Stokes and pump lights are parallelly aligned during propagation. $L_{eff}$ and $A_{eff}$ represent the effective length and effective core area of the optical fiber, respectively. Therefore, the threshold peak power can be controlled by adjusting the core size and length of the optical fiber, and the power required for the SRS can be controlled. When the power of the generated Stokes light exceeds $P_{th}$, a subsequent higher-order Stokes Raman process can be initiated. The efficiency and spectral output of SRS are also significantly influenced by the pulse width and PRR of the pump laser. A shorter pulse width increases the peak power for a given average power, thereby enhancing the nonlinear interaction and enabling more efficient generation in high-order Stokes components. Conversely, a higher PRR facilitates high-speed image acquisition in OR-PAM but may reduce pulse energy, leading to a decrease in peak power and limiting Raman gain. Therefore, optimizing the trade-off between pulse width and PRR is essential to maximize both SRS efficiency and imaging speed.

The length of the optical fiber also affects SRS performance. Shorter fibers increase the difficulty of generating higher-order Stokes lights due to the reduced nonlinear interaction lengths. However, they provide a wider usable pulse energy range for lower-order Stokes generation, which is beneficial for stable operation at higher pulse energies. In contrast, longer optical fibers facilitate the generation of multiple higher-order Stokes wavelengths by extending the interaction length, thereby enhancing wavelength tunability. Therefore, the fiber length should be carefully optimized to balance pulse energy and wavelength coverage, depending on the specific requirements of applications.

Raman gain within the range of 11–15 THz is produced in the frequency-shifted region when a pump light with an intensity exceeding the critical peak power propagates through the Raman fiber. Without an external seed light, the wavelength corresponding to a Raman shift of ~13.2 THz, which exhibits the highest gain in the Raman gain spectrum, is preferentially amplified. Wavelength generation can be achieved even in spectral regions lacking conventional gain materials by leveraging the cascaded SRS phenomenon in Raman fibers, wherein high-order Stokes light is generated while maintaining a constant Raman shift. Multiwavelength SRS light sources based on the cascaded SRS process have been extensively researched as light sources for multispectral OR-PAM in both the visible and near-infrared (NIR) regions.

## 3. SRS Light Source in the Visible Spectral Region

The visible spectrum (400–700 nm) is critical for OR-PAM because of the strong optical absorption of endogenous chromophores such as HbO and HbR [76]. Hemoglobin exhibits distinct optical absorption peaks in this range, which makes multispectral illumination essential for accurate functional imaging. However, the direct generation of multiwavelength light sources in the visible range is challenging because many wavelengths lack a corresponding gain medium for efficient amplification.

To overcome this challenge, SRS has been widely utilized for generating multiwavelength light sources in the visible range. The SRS light source enables the generation of multiple discrete Stokes wavelengths by employing a 532 nm nanosecond pulsed laser as a pump light, which extends to ~700 nm [77]. This technique facilitates access to wavelengths that are otherwise difficult to generate because of the lack of an appropriate gain medium. SRS light sources are advantageous for enhancing sO_2_ mapping and blood vessel visualization because hemoglobin exhibits strong absorption within the 500–600 nm range. In early studies, SRS light sources for OR-PAM were generated using the conventional approach that employs a single Raman fiber [78,79]. This method enables producing multiwavelength SRS light sources through a relatively simple optical setup. Specific orders of Stokes lights can be selectively obtained by adjusting the Raman fiber length and the power of the pump light. Most studies employing this method use spectral filters to selectively isolate different Stokes wavelengths before PAI. This technique has the advantage of distinguishing different biological tissues based on their optical absorption characteristics.

**Figure 4:**
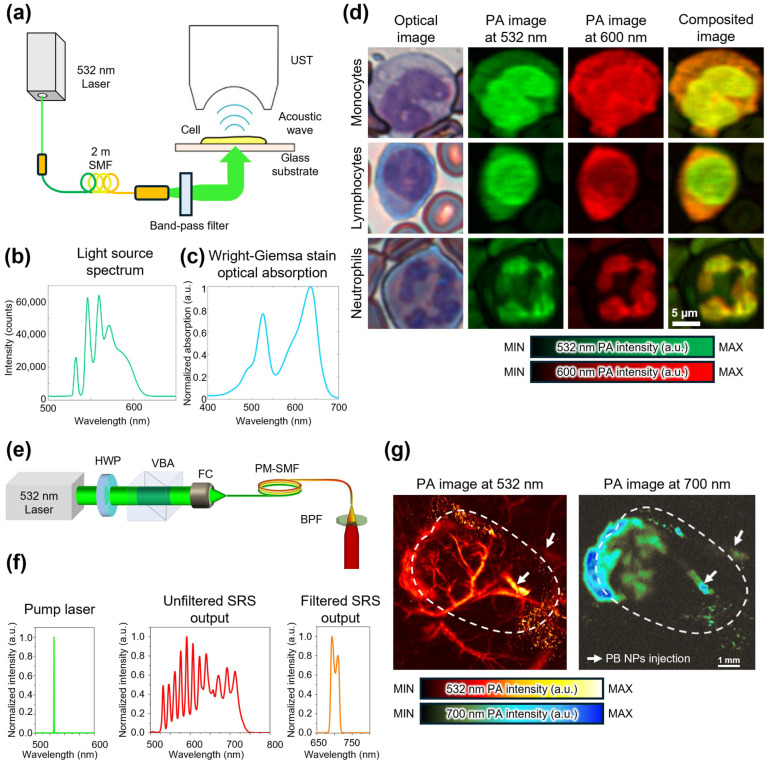
(**a**) Schematic of OR-PAM employing an SRS light source for blood smear detection. (**b**) Spectrum of the SRS output. (**c**) Optical absorption spectrum of the Wright–Giemsa stain. (**d**) Optical image and multispectral PA images of neutrophils, lymphocytes, and monocytes. (**e**) Schematic of OR-PAM employing an SRS light source for blood and PB NP detection. (**f**) Spectrum of pump light, unfiltered and filtered SRS output. (**g**) Multispectral PA images of the mouse ear tumor model at 532 and 700 nm wavelengths. The dash lines indicate the tumor region. OR-PAM, optical-resolution photoacoustic microscopy; SRS, stimulated Raman scattering; PA, photoacoustic; PB NPs, Prussian blue nanoparticles; SMF, single-mode fiber; UST, ultrasound transducer; HWP, half-wave plate; VBA, variable beam splitter/attenuator; FC, fiber coupler; PM-SMF, polarization-maintaining single-mode fiber; BPF, band-pass filter. The images are reproduced with permission from Refs. [80,81].

Strohm et al. used an OR-PAM system that employs an SRS light source to obtain labeled cell images (Figure 4a) [80]. A 2-m-long single-mode fiber (SMF) used as the Raman fiber in the SRS light source generates multiple discrete Stokes lights with a broad wavelength of 545–620 nm using a 532 nm pump light (Figure 4b). The SRS output has a broad bandwidth, and therefore, a band-pass filter (BPF) is used to select specific wavelengths, ultimately choosing 532 and 600 nm wavelengths for imaging Wright–Giemsa-stained blood smears. Several dyes, including Eosin Y and methylene blue in the Wright–Giemsa stain, exhibit different optical absorption coefficients at wavelengths of 532 and 600 nm (Figure 4c). These dyes are selectively absorbed in various organelles, which enables distinguishing them. Consequently, different PA signal intensities are obtained for each cellular organelle using varying light sources. Strohm et al. used multispectral PAI with an SRS light source to effectively distinguish between the nuclei and cytoplasms of neutrophils, lymphocytes, and monocytes (Figure 4d). Bui et al. synthesized Prussian blue nanoparticles (PB NPs), which possess unique optical absorption properties that distinguish them from blood cells, and imaged them in vivo using an OR-PAM system by employing an SRS light source (Figure 4e) [81]. They utilized two types of optical fibers in the SRS light source: a 2-m-long SMF for the 532 nm PAI and a 35-m-long polarization-maintaining single-mode fiber (PM-SMF) as the Raman fiber for the 700 nm PAI. The PM-SMF generates Stokes light via the SRS process using a 532 nm pump light with a PRR of 5 kHz, which produces an output spectrum extending up to 712 nm. This wavelength range is sufficient for exciting PB NPs, which have maximum absorption at 700 nm. For the 700 nm PA image, the SRS output is filtered by a BPF with a center wavelength of 700 nm and bandwidth of 25 nm (Figure 4f). They acquired multispectral images of the mouse ear tumor model using wavelengths of 532 and 700 nm (Figure 4g). In the 532 nm PA image, both blood vessels and PB NPs were detected, and the blood vessel structure was clearly visible. In contrast, at 700 nm, the blood vessel structure disappeared from the PA image because of the rapid decline in the hemoglobin optical absorption coefficient beyond 600 nm. However, the distribution of the PB NPs remained clearly observable owing to their high optical absorption coefficients at 700 nm.

**Figure 5:**
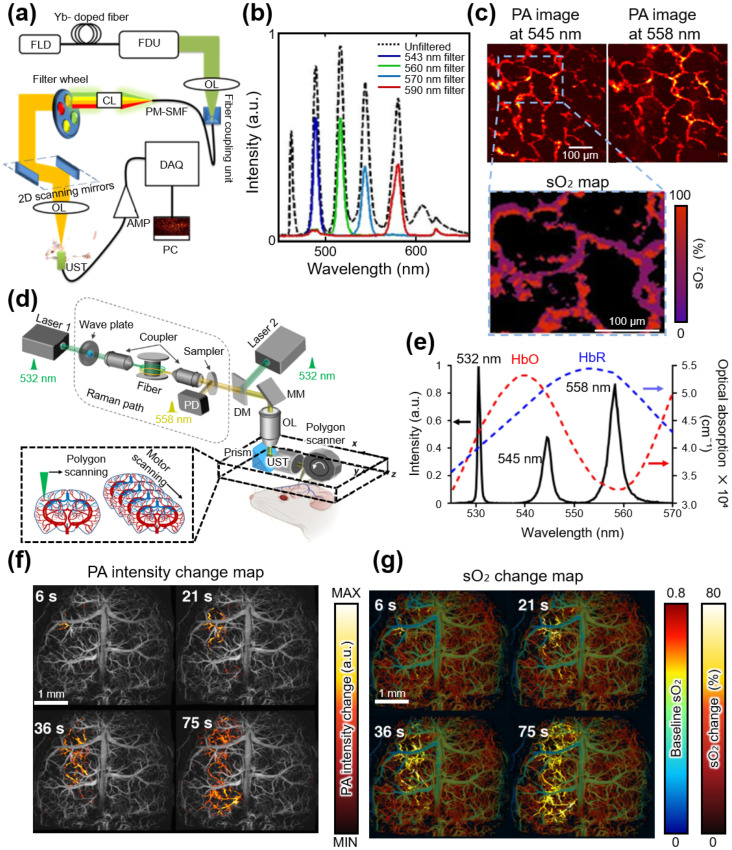
(**a**) Schematic of OR-PAM employing an SRS light source for functional PAI. (**b**) Spectrum of the SRS output with and without a filter. (**c**) PA images of the mouse ear at wavelengths of 545 and 558 and the sO_2_ map. sO_2_ can be calculated by spectral unmixing. (**d**) Schematic of real-time multispectral OR-PAM employing an SRS light source, which includes an additional 532 nm laser. (**e**) Combined output spectrum of the 532 nm laser and SRS light source, along with the optical absorption spectra of HbO and HbR. Each graph follows the vertical axis labels with arrows of the same color. (**f**) Real-time PA intensity change maps and (**g**) sO_2_ change maps associated with the propagation of spreading depolarization waves in the mouse brain. OR-PAM, optical-resolution photoacoustic microscopy; SRS, stimulated Raman scattering; PAI, photoacoustic imaging; PA, photoacoustic; sO_2_, oxygen saturation; HbO, oxy-hemoglobin; HbR, deoxy-hemoglobin; FLD, fiber laser diode; FDU, frequency-doubling unit; OL, objective lens; PM-SMF, polarization-maintaining single-mode fiber; CL, collimator lens; UST, ultrasound transducer; AMP, amplifier; DAQ, data acquisition; MM, mirror; SMF, single-mode fiber; PD, photodiode; DM, dichroic mirror. The images are reproduced with permission from Refs. [82,83].

Some studies acquired functional information from multispectral PAI using an SRS light source. For example, Hajireza et al. acquired a functional information map using an OR-PAM with an SRS light source and a spectral unmixing method (Figure 5a) [82]. They generated multiple discrete Stokes wavelengths extending up to the fifth-order Stokes wavelength at 600 nm using a 532 nm pump light with a 1 ns pulse duration and a PM-SMF. In addition, they employed a filter wheel to actively select the wavelength of the SRS output. The wavelength of the filtered SRS output was adjusted to 543, 560, 570, or 590 nm by selecting BPFs with different center wavelengths (Figure 5b). Further, they optimized the conditions of the SRS light source by varying the length of the PM-SMF and PRR of the pump laser. They generated the SRS output via a 40 kHz PRR pump light and a 3-m-long PM-SMF subsequently employed for in vivo functional PAI to minimize four-wave mixing (FWM) and maintain optimal pulse energy levels. After acquiring multispectral PA images at 532 nm and 558 nm wavelengths, sO_2_ maps were derived through the spectral unmixing method (Figure 5c). However, SRS light sources utilizing advanced BPFs inherently experience slight movements when the spectral filters are changed. This results in spatial misalignment between images, necessitating additional postprocessing steps for accurate multispectral analysis. In addition, the time delay between wavelength adjustment and the reacquisition of PA images can introduce errors caused by motion artifacts.

To address these issues, recent studies actively explored methods for multispectral PA imaging that do not require filter changes. Zhu et al. acquired multispectral PA images by alternately oscillating two lasers without changing the filters (Figure 5d) [83]. They employed two 532 nm lasers, with one integrated into an SRS light source for adjusting the wavelength of OR-PAM. A 6.5-m-long PM-SMF was utilized as the Raman fiber, which generated second-order Stokes light at 558 nm from a 532 nm pump laser. The 532 and 558 nm lights were combined using a dichroic mirror (Figure 5e). A polygon scanner enabled a scanning area of 11 × 7.5 mm^2^ with a scan speed of 2 Hz, and their system successfully acquired real-time PA images and sO_2_ changes associated with spreading depolarization (SD) waves in a mouse brain (Figure 5f,g). By incorporating additional lasers, the SRS light source does not require filter replacement, which enables high-speed multispectral PAI.

To obtain multispectral PA images with rapid wavelength switching using a single pump laser, a recent study proposed a multipath approach that splits the pump laser into separate paths, each independently inducing an SRS prior to recombination [84,85,86]. The optimal Stokes wavelength for each path can be precisely designed by configuring each path with a different length of Raman fiber and peak power of the pump light, and the time delay between the pulses in each path can be controlled. These independent SRS channels enable generating multispectral PA signals within a single A-line using a single pump laser. Moreover, this approach eliminates the need for filter switching to change the wavelength during imaging, enhancing both the spatial and temporal accuracies of the multispectral PA images.

**Figure 6:**
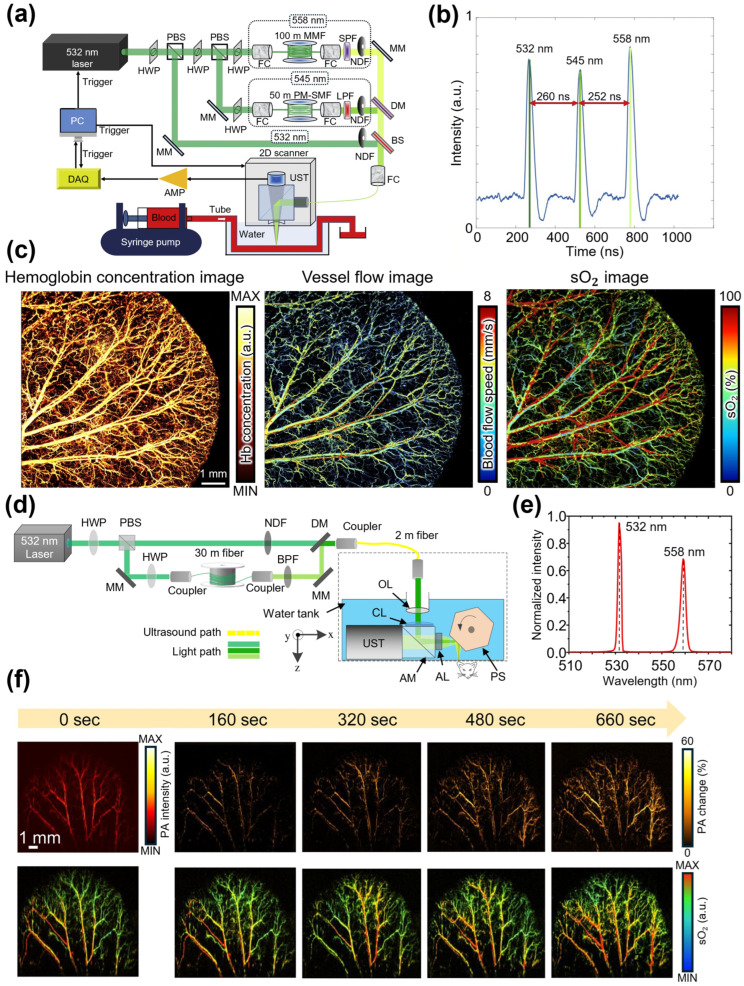
(**a**) Schematic of OR-PAM employing a multipath SRS light source for multiple functional PAI. (**b**) Time trace of SRS output. (**c**) Multiple functional PA images of hemoglobin concentration, blood flow, and compensated sO_2_. (**d**) Schematic of real-time OR-PAM employing a multipath SRS light source. (**e**) Spectrum of the SRS output. (**f**) Time-lapse PA images are used to monitor PA intensity and hematological changes in the mouse ear after epinephrine administration. OR-PAM, optical-resolution photoacoustic microscopy; SRS, stimulated Raman scattering; PAI, photoacoustic imaging; PA, photoacoustic; sO_2_, oxygen saturation; AMP, amplifier; MM, mirror; DM, dichroic mirror; DAQ, data acquisition; FC, fiber coupler; HWP, half-wave plate; LPF, long-pass filter; MMF, multimode fiber; PBS, polarizing beam splitter; PM-SMF, polarization-maintaining single-mode fiber; SPF, short-pass filter; UST, ultrasound transducer; NDF, neutral density filter; BPF, band-pass filter; CL, correction lens; AM, acoustic mirror; AL, acoustic lens; PS, polygon scanner. The images are reproduced with permission from Refs. [85,87].

Liu et al. obtained multiple functional PA images in a single C-scan and enhanced the accuracy of sO_2_ maps using OR-PAM by employing a multipath SRS light source [85]. The Grüneisen relaxation effect is an important factor to consider in functional PAI. The pulse energy may be delivered before chromophores are fully relaxed when a light source operates at an excessively high PRR, potentially causing errors in the PA signal intensity. Therefore, a high-PRR light source must account for these effects to ensure accurate functional information measurements. In this study, multispectral PA images were acquired at three wavelengths to extract functional information and compensate for sO_2_ estimation errors. A multipath SRS light source was used to generate a three-wavelength light source (Figure 6a). A beam splitter splits the pump light with a wavelength of 532 nm and duration of 7 ns into three paths. In the 532 nm path, the pump light propagated through free space without fiber coupling. In the 545 nm path, a Stokes wavelength of 545 nm was generated using a 50-m-long PM-SMF and filtered using a 540 nm long-pass filter. Finally, in the 558 nm path, a 100-m-long multimode fiber generated a Stokes wavelength of 558 nm, which was filtered using a 570 nm short-pass filter. Each beam was subsequently combined into a single optical path and delivered through an optical fiber for scanning. The pulses of each wavelength experienced temporal delays of ~250 ns each because of the differences in the optical path lengths (Figure 6b). Using this multiwavelength SRS light source, the hemoglobin concentration, blood flow, and corrected sO_2_ maps of the mouse ear were successfully acquired in a C-scan (Figure 6c).

Chen et al. developed OR-PAM by employing a multipath SRS light source at wavelengths of 532 and 558 nm. The SRS light source enabled multispectral PAI at a frame rate of 1 frame per second (fps) over a scanning area of 12 × 5 mm^2^ (Figure 6d) [87]. The third-order Stokes light at 558 nm, generated from a 30-m-long PM-SMF Raman fiber, was irradiated with a 150 ns delay relative to the 532 nm wavelength because of the length of the Raman fiber (Figure 6e). The pulses of each wavelength were combined into a single path using a dichroic mirror, and the combined SRS output was used for PAI with a polygon-motor-integrated scanner. This system enabled rapid scanning using the 1 MHz PRR of the pump laser, and it was used to image a mouse ear in 20 s cycles, monitoring changes following intravenous epinephrine injection (Figure 6f).

**Figure 7:**
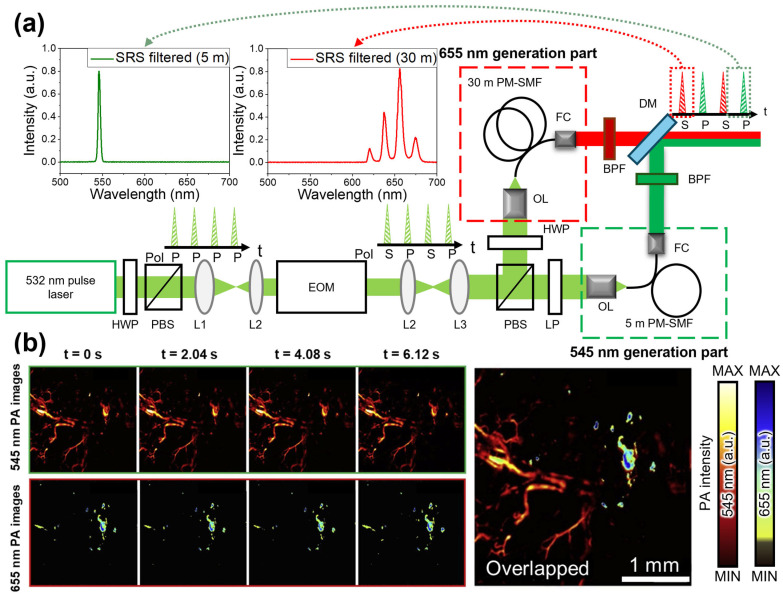
(**a**) Schematic of real-time OR-PAM employing a polarization-modulated SRS light source. This OR-PAM can provide a wide wavelength range of 650 nm or more because it can use almost all of the preserved pump light power. (**b**) Time-lapse PA images at 545 and 655 nm of a mouse ear. OR-PAM, optical-resolution photoacoustic microscopy; SRS, stimulated Raman scattering; PA, photoacoustic; PBS, polarization beam splitter; L, lens; EOM, electro-optic modulator; OL, objective lens; FC, fiber collimator; BPF, band-pass filter; DM, dichroic mirror; HWP, half-wave plate; LP, linear polarizer; PM-SMF, polarization-maintaining single-mode fiber. The images are reproduced with permission from ref. [88].

Despite these advancements, multipath SRS light sources still face several challenges. The primary limitation is the division of the pump peak power across multiple paths, which reduces the available peak power per path and restricts the generation of higher-order Stokes light. Multiwavelength SRS light sources based on polarization modulation have been proposed to address this issue. For example, Park et al. developed an OR-PAM employing a polarization-modulated SRS light source that effectively overcame the limitations of a multipath SRS light source [88]. In their approach, the polarization of a 532 nm pump light with a 300 kHz PRR was modulated using an electro-optic modulator (EOM), alternately switching between p- and s-polarizations. Subsequently, the modulated light was split using a polarizing beam splitter (PBS) and directed into two separate SRS paths (Figure 7a). Nearly 90% of the pump laser power was preserved in each path, enabling efficient generation of high-order Stokes light beyond 600 nm. To target specific wavelengths, two Raman fibers of different lengths (5 m and 30 m) were used, with each fiber having distinct critical peak powers for optimal Stokes generation. BPFs at 545 nm (for the 5 m path) and 655 nm (for the 30 m path) were used to isolate the desired wavelengths. The two Stokes light beams were then combined into a single optical path using a dichroic mirror. The polarization-modulated SRS light source, emitting at 545 nm and 655 nm, was used to acquire multispectral PA images at a frame rate of 0.5 fps (Figure 7b). These results demonstrated that the spatial distributions of blood vessels and gold nanorods could be effectively distinguished by overlaying the PA images acquired at two different wavelengths.

## 4. SRS Light Source at the Near-Infrared Spectral Region

Recent research has focused on SRS light sources in the NIR region to extend the capabilities of PAI beyond the visible region. Studies in this region primarily aim at generating wavelengths corresponding to the first and second overtones of C-H bonds, particularly around 1200 and 1700 nm. C-H bonds, which are the fundamental building blocks of all organic compounds, exhibit distinct vibrational modes in this spectral region, which enables chemical-specific imaging. The ability to analyze spectral information within this range facilitates the effective differentiation of various organic substances [89]. This approach can be applied to target lipids, which are abundant C-H bond biomarkers, for imaging lipid-rich tissues such as atherosclerotic plaques, myelinated nerve fibers, and coronary artery plaques. OR-PAM, which employs SRS light sources capable of generating wavelengths in the 1200 nm and 1700 nm spectral bands, was developed to detect various organic compounds containing C-H bonds.

Wilkinson et al. proposed OR-PAM employing an SRS light source for imaging lipid-rich tissues [90]. They utilized the output of a Q-switched 1064 nm Nd microchip laser as the pump light, which has a pulse duration of 0.6 ns and a PRR of 7.4 kHz. A 20-m-long SMF was used as the Raman fiber to generate the SRS. Subsequently, the SRS output was filtered using BPFs centered at 1064, 1100, 1175, 1225, 1275, and 1325 nm. Spectroscopic PA images acquired from lipid phantoms demonstrated high contrast at 1175 and 1225 nm, which are wavelengths near 1210 nm, where lipids exhibit strong optical absorption. This study demonstrated that an SRS light source can effectively generate wavelengths for efficient lipid detection. However, compared to 532 nm, the 1064 nm pump wavelength exhibited a broader Raman gain bandwidth (exceeding 10 nm), resulting in progressive spectral broadening and reduced energy density with the generation of a higher-order Stokes light. Consequently, spectral filters with broad bandwidths were frequently used to ensure sufficient PA signals. Although this approach increased the signal strength, it limited the ability to obtain high-resolution spectroscopic PA information within narrow wavelength bands.

**Figure 8:**
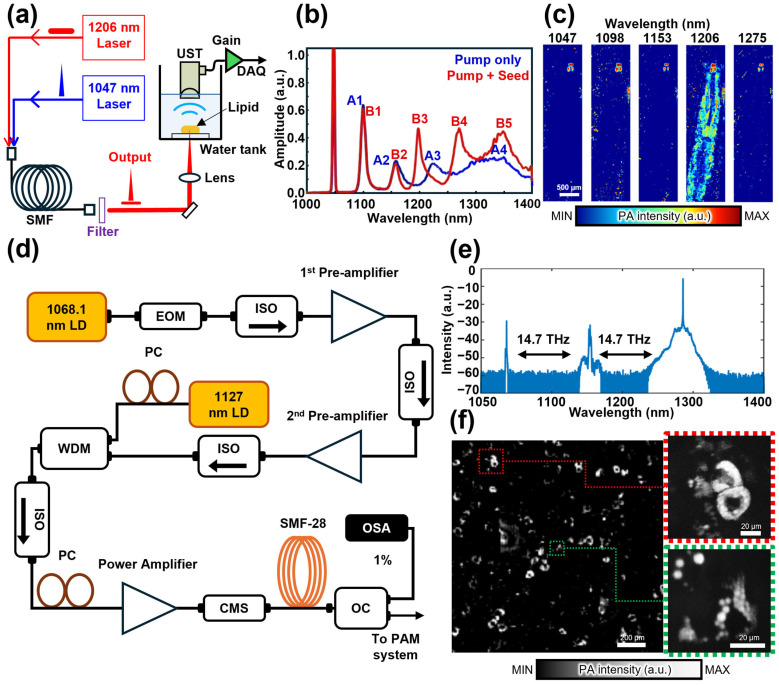
(**a**) Schematic of injection-seeded SRS light source for generating narrow Stokes lights. (**b**) Spectrum of SRS output generated by the injection-seeded SRS light source. (**c**) Multispectral PA images of drosophila larva at wavelengths in the range of 1047–1275 nm. (**d**) Schematic of injection-seeded SRS light source using FWM-based seed light (**e**) Spectrum of the SRS output generated by the injection-seeded SRS light source. (**f**) PA images of human white adipocytes and Zoom-ins. SRS, stimulated Raman scattering; PAM, photoacoustic microscopy; UST, ultrasound transducer; DAQ, data acquisition; SMF, single-mode fiber; LD, laser diode; EOM, electro-optic modulator; ISO, isolator; WDM, wave division multiplexer; PC, polarization controller; CMS, cladding mode stripper; OC, Optical coupler; OSA, optical spectrum analyzer. The images are reproduced with permission from Refs. [91,92].

Choi et al. applied an injection-seeding technique to overcome the limitations of fixed wavelengths and broad spectral bandwidths in previous OR-PAM systems (Figure 8a) [91]. To this end, they combined a 1047 nm Q-switched Nd: YLF pump light operating with a 14 ns pulse duration and a 2.5 kHz PRR with a 1206 nm multimode diode seed light featuring a 2 μs pulse duration and the same PRR. Both the pump and seed lights, which were polarized parallel to each other, were delivered through a 100-m-long SMF. The 1206 nm seed light was not directly amplified by the 1047 nm pump light, and instead, it was amplified by the second-order Stokes light generated in the Raman fiber. The spectra of the third- and fourth-order Stokes light significantly narrowed when the seed light was used (Figure 8b). The SRS output was filtered through a BPF. A 10 nm bandwidth filter was used at 1050 nm, while broader filters with 50 nm bandwidths were employed for other wavelengths, including 1100, 1150, 1225, and 1275 nm. They validated OR-PAM by employing an injection-seeded SRS light source and acquiring multispectral PA images of a Drosophila larva (Figure 8c). The multispectral PA images showed the maximum intensity at 1206 nm, which was close to the expected lipid absorption peak at 1210 nm. These results demonstrated that the spectral broadening of higher-order Stokes light, which was commonly observed in conventional SRS light sources, could be effectively suppressed using seed light.

Lee et al. presented an SRS amplifier using an FWM-based injection seeding technique (Figure 8d) [92]. In general, wavelength selectivity in an SRS light source is achieved using BPFs. In their study, both the wavelength selectivity and Raman conversion efficiency of the SRS source were enhanced by employing an FWM-based seed light. Further, they used a narrow-linewidth distributed feedback laser diode (DFB-LD) operating at 1068.1 nm as the pump laser and measured the total gain through the two preamplifiers, which was approximately 25 dB. Each preamplifier was combined with a BPF with a 2 nm bandwidth centered at 1064.1 nm to perform filtering. Further, Raman fibers were implemented using an SMF. The seed light operating at a center wavelength of 1130 nm suppressed the spectral broadening of the Raman gain via FWM and fine-tuned the Raman shift frequency. The seed light adjusted the Raman shift to 14.7 THz instead of the typical 13.2 THz (Figure 8e), and through FWM, the third-order Stokes light was shifted to 14.7 THz, generating Stokes lights. The spectra of the Stokes lights produced a 1127 nm wavelength for the first-order Stokes light and a 1192.8 nm wavelength for the second-order Stokes light, which corresponds to a Raman shift of 14.7 THz. At the peak power observed at 1192.8 nm (second-order Stokes light), the pulse energy, bandwidth, pulse duration, and PRR were 5 μJ, 70 pm, 2 ns, and 200 kHz, respectively. The OR-PAM employing an injection-seeded SRS light source was validated using a PA image of human white adipocytes (Figure 8f). The acquired PA images demonstrated a high resolution that could resolve lipid droplets as small as 3.2 µm. Further, a signal-to-noise ratio (SNR) of 27.1 dB was achieved in the white adipocytes. The key contribution of this study lies in adjusting the seed light via FWM to modify the Raman shift frequency, which shifts the dominant Raman shift from 13.2 THz to 14.7 THz, creating narrow linewidth Stokes light at the desired Raman shift interval. Thus, the OR-PAM utilizing an injection-seeded SRS light source in the NIR region is an efficient method for generating diverse light sources for imaging lipid-rich tissues. Ongoing research is focused on controlling the wavelength of the Stokes light, and this is expected to help enhance the wavelength selectivity of the generated Stokes light and improve the quality of the PA images.

**Figure 9:**
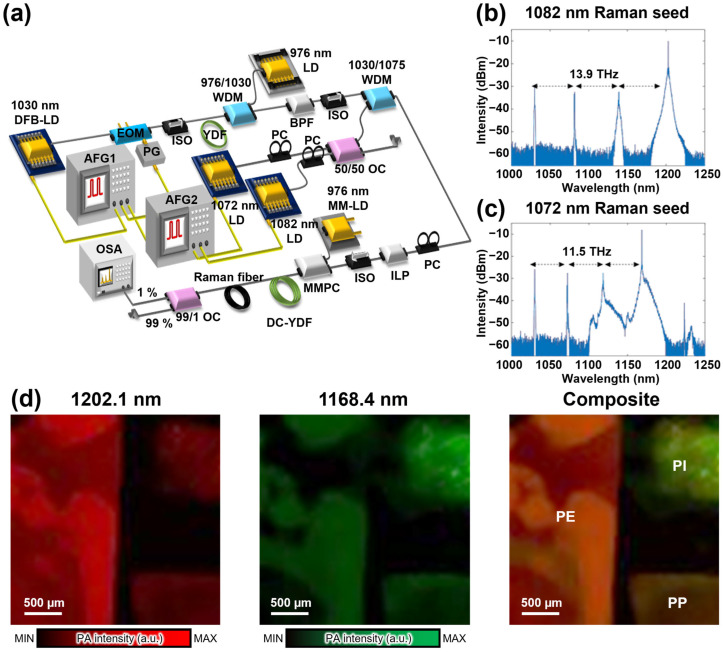
(**a**) Schematic of the dual-wavelength SRS light source using the FMW-based seed light for the OR-PAM system. Spectra of the SRS output with (**b**) 1082 and (**c**) 1072 nm seed lights. (**d**) Multispectral PA images of PE, PI, and PP at wavelengths of 1202.1 and 1168.4 nm. SRS, stimulated Raman scattering; FWM, four-wave mixing; OR-PAM, optical-resolution photoacoustic microscopy; PA, photoacoustic; PE, polyethylene; PI, polyimide; PP, polypropylene; AFG, arbitrary function generator; BPF, band-pass filter; DC-YDF, double-clad ytterbium-doped fiber; EOM, electro-optic modulator; ILP, in-line polarizer; ISO, isolator; LD, laser diode; MM-LD, multimode laser diode; MMPC, multimode pump combiner; OC, optical coupler; OSA, optical spectrum analyzer; PC, polarization controller; PG, pulse generator; WDM, wavelength division multiplexer; YDF, ytterbium-doped fiber. The images are reproduced with permission from ref. [93].

Recently, significant progress has been achieved in overcoming the limitations of discrete wavelength tuning in SRS light sources within the NIR region, which has enabled more accurate acquisition of spectroscopic PA information. For example, Lee et al. introduced an electronically controlled dual-wavelength SRS light source using FWM-based seed light operating in the NIR-II region [93] (Figure 9a), specifically targeting 1168.4 nm. This SRS light source achieved rapid wavelength switching through electronic control and nanosecond pulse durations. The SRS light source generated Stokes light with Raman shifts of 13.9 (Figure 9b) and 11.5 THz (Figure 9c) by manipulating dual-wavelength seed light, which helped expand the wavelength tuning range. This SRS light source was based on a master oscillator fiber amplifier seeded by a 1030 nm DFB-LD, which initially generated 10 ns pulses that were refined into 2 ns pulses using an EOM. Subsequently, the pump light was amplified using a single-mode ytterbium-doped fiber amplifier (YDFA) and coupled with two seed DFB-LDs operating at 1072.3 and 1081.6 nm, achieving spectral narrowing through FWM and SRS amplification. Further, the amplified pump light was directed into a double-clad YDFA pumped by a 976 nm laser diode, which enhanced its power. The amplified pump light was coupled into a Raman fiber composed of a 2-m-long SMF-28 and a 3-m-long HI-1060 fiber to generate Stokes light. The SRS output achieved pulse energies, pulse durations, and a repetition rate of 3 µJ, 2 ns, and 200 kHz, respectively, with rapid wavelength switching at 100 kHz. The synchronized electronic modulation achieved dual-wavelength emission at 1168.4 and 1202.1 nm, enabling efficient multispectral PAI and improving the differentiation of organic materials based on their absorption spectrum. The validation of this SRS light source was conducted through PA images of three different polymer films (Figure 9d), with each image acquired using an average of 200 scans. The OR-PAM utilized wavelengths of 1202.1 and 1168.4 nm over a field of view (FOV) of 3 × 3 mm^2^. In addition, imaging the polymer films revealed higher absorption in polyethylene (PE) at 1202.1 nm and in polyimide at 1168.4 nm, exhibiting a clear distinction between the materials. The system demonstrated the ability to image polymer films such as PE, polypropylene (PP), and polyimide (PI), which exhibited distinct C-H absorption peaks.

**Figure 10:**
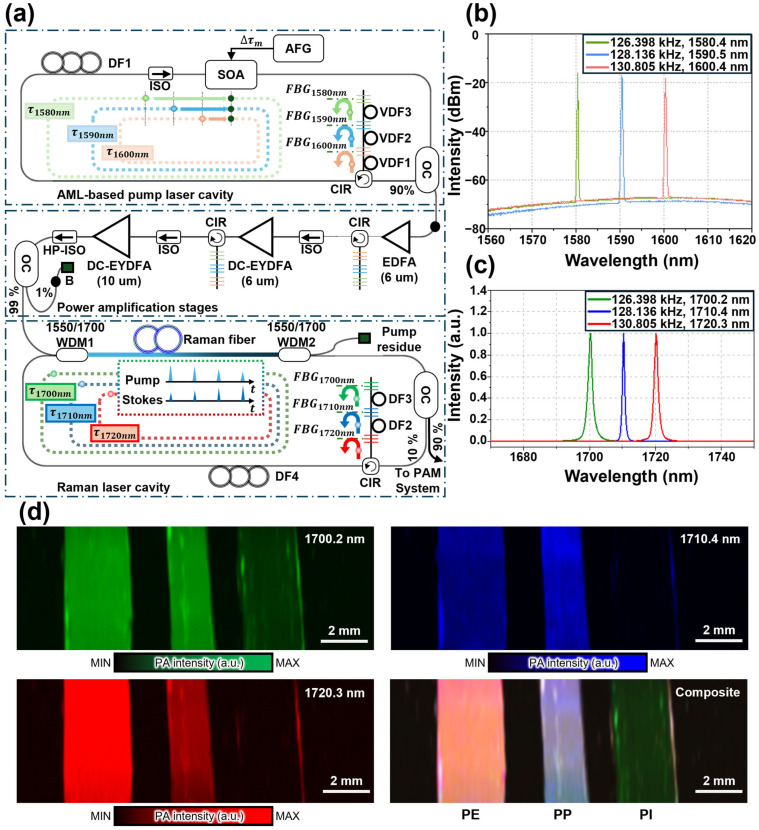
(**a**) Schematic of synchronously pumped SRS light source for OR-PAM. (**b**) Spectrum of the pump light at modulation frequencies of 126.398, 128.136, and 130.808 kHz, which correspond to wavelengths of 1580.4, 1590.5, and 1600.4 nm, respectively. The colored arrows indicate the direction in which light progress. (**c**) Spectrum of SRS output corresponding to modulation frequency variations at 126.398, 128.136, and 130.808 kHz, with pump light wavelengths of 1580.4, 1590.5, and 1600.4 nm, respectively. (d) Multispectral PA images of PE, PP, and PI films at wavelengths of 1700.2, 1710.4, and 1720.3 nm, and composite. SRS, stimulated Raman scattering; OR-PAM, optical-resolution photoacoustic microscopy; PA, photoacoustic; PE, polyethylene; PP, polypropylene; PI, polyimide; AFG, arbitrary function generator; SOA, semiconductor optical amplifier; ISO, isolator; FBG, fiber Bragg grating; DF, delayed fiber; VDF, variable delayed fiber; CIR, circulator; OC, optical coupler; EDFA, erbium-doped fiber amplifier; DC-EYDFA, double-clad erbium/ytterbium-doped fiber amplifier; HP-ISO, high-power isolator; WDM, wavelength division multiplexer. The images are reproduced with permission from ref. [94].

Similarly, Park et al. developed a wavelength-switchable, synchronously pumped SRS light source operating in the 1700 nm region, which is crucial for detecting the first overtone of C-H bonds [94]. Unlike previously reported SRS light sources in the 1200 nm region, there is no readily available seed laser at 1700 nm. To address this limitation, the wavelength of the Raman gain generated within the Raman fiber was utilized as a seed light by constructing a Raman cavity that incorporates multiple fiber Bragg grating (FBG) segments. This SRS light source employs a dual-cavity design that synchronizes the round-trip times of both the pump and seed lights, thereby enabling active wavelength switching among 1700.2, 1710.4, and 1720.3 nm (Figure 10a). In this design, the pump light uses an active mode-locking technique with a semiconductor optical amplifier as the gain medium. The pump light is modulated to generate nanosecond pulses, and the wavelength is switched to 1580.4, 1590.5, and 1600.4 nm (Figure 10b) by matching the modulation frequency to the free spectral range (FSR) of the pump laser cavity. The pump light is amplified using one erbium-doped and two double-clad erbium/ytterbium-doped fiber amplifiers, which boost the pulse energy beyond 1 µJ. The amplified pump light is coupled into a Raman laser cavity using wavelength-division multiplexers. The first-order Stokes light achieves the highest gain at 1700, 1710, and 1720 nm when the pump wavelength is switched to 1580, 1590, and 1600 nm, respectively, which corresponds to a Raman shift of 13.2 THz for each pump wavelength (Figure 10c). The Raman laser cavity uses different FBG arrays to match the FSR of Stokes lights with pump light wavelengths, thereby satisfying synchronous pumping conditions. The SRS output spectrum is electrically controlled by adjusting the modulation frequency of the pump light, which enables switching to 1700.2, 1710.4, and 1720.3 nm with modulation frequencies of 126.398, 128.136, and 130.805 kHz, respectively. The SRS output pulse energy exceeded 500 nJ, with a pulse duration of ~4 ns. The PA images were acquired with an FOV of 15 × 5 mm^2^ in the x–y plane using a scanning step size of 10 μm in the x direction and 50 μm in the y direction (Figure 10d). The three polymers (PI, PP, and PE) exhibited the strongest PA signals at 1700.2, 1710.4, and 1720.3 nm, respectively. The composite image formed by combining the PA images at the three different wavelengths clearly distinguished the three polymer types. Integrated into a custom OR-PAM system, this synchronously pumped SRS light source facilitated spectroscopic PA imaging of PE, PP, and PI films based on their distinct C-H bond absorption characteristics. The flexibility of tuning the Raman gain and switching the seed wavelength shows significant potential for the spectroscopic PA analysis of molecules with different structural groups in the overtone region of the C-H bond.

## 5. Discussion and Conclusions

OR-PAM is an advanced imaging modality that enables high-contrast visualization of both endogenous and exogenous contrast agents with optical resolution by leveraging the PA effect. This technique facilitates the noninvasive monitoring of metabolic processes and biological responses in vivo. OR-PAM has been extensively employed in preclinical investigations and emerging clinical applications because of its unique capability of combining high spatial resolution with functional imaging depth.

In contrast to the conventional single-wavelength PAI, multispectral PAI enhances the differentiation of contrast agents by exploiting their distinct optical absorption spectra. Further, it enables the extraction of additional quantitative and functional information through mathematical approaches such as spectral unmixing. Employing a light source that can deliver a high PRR, multiple discrete wavelengths, short pulse durations, and sufficient pulse energy is essential to realize a multispectral OR-PAM system with continuous monitoring and high SNR. In this context, SRS light sources are explored to overcome the inherent limitations of commonly used OPOs and supercontinuum sources.

This review summarizes recent advances in the development of SRS light sources for multispectral PAI in the visible and NIR spectral regions (Table 1). In the visible region, SRS light sources have been utilized to generate functional PAI signals from chromophores such as hemoglobin, which exhibit strong absorption between 500 and 600 nm. These light sources have been widely applied in functional imaging tasks, including chromophore identification, oxygen saturation mapping, and blood flow measurement, and demonstrated notable utility in the continuous vascular imaging of small animals. However, conventional SRS light sources require the manual replacement of spectral filters to switch between wavelengths, introducing temporal and spatial inconsistencies that limit the feasibility of real-time functional PAI.

To address this limitation, advanced configurations using multipath or polarization-modulated techniques have been developed to acquire multiwavelength PA images within a single scan. In the NIR region, significant research efforts have been devoted to generating wavelengths of ~1200 and 1700 nm, corresponding to the first and second overtones of the C-H bonds, respectively. As C-H bonds are the fundamental structural components of most organic molecules, targeting these wavelengths enables the effective discrimination of substances such as lipids, collagen, and synthetic polymers. However, in the NIR region, the SRS light source has a wide Raman gain range of several tens of nanometers, which is a problem that does not commonly occur in the visible region. Injection seeding and synchronous pumping techniques have been employed to overcome this problem. This approach employs a seed light at wavelengths within the broadband Raman gain region to produce SRS outputs characterized by narrow spectral linewidths and enhanced wavelength selectivity. In addition, this technique facilitates multispectral PAI acquisition in the NIR region by enabling the dynamic switching of the Raman output wavelength through the modulation of the seed light wavelength. This advancement significantly enhances spectral resolution and tunability, which enables more accurate and flexible spectral analysis within the Raman gain bandwidth. In addition, by integrating advanced high-speed scanning mechanisms [95], the application scope of SRS sources in PAI could be further expanded, as the current PRR limitations are largely dictated by the scanning speed rather than the laser source itself.

While OR-PAM systems utilizing SRS light sources have demonstrated significant potential for multispectral PAI across both the visible and NIR spectral regions, several critical considerations must be addressed to ensure optimal performance. One key factor is the stability of the light source. The nonlinear characteristics of SRS can be influenced by environmental factors such as temperature fluctuation, airflow, and time [96]. Although laser stability generally improves at lower temperatures, the standard deviation of average power can be reduced to below 3% through careful optimization of system parameters, such as the polarization state of the pump beam. Additionally, variations in pulse energy can be compensated using photodiode-based energy monitoring and correction techniques [97,98].

Another crucial consideration is the efficient coupling of the pump laser into the Raman fiber to achieve the necessary peak power for effective SRS generation. Recent studies have demonstrated the feasibility of automated laser–fiber coupling techniques [99,100], which can mitigate issues such as minor misalignments, mode mismatches, and beam quality degradation. These approaches contribute to enhanced output stability and more reliable multispectral performance in OR-PAM systems.

An SRS light source is currently applied to OR-PAM due to its insufficient output energy for other PAI systems such as PACT, where light delivery in deep-tissue is required [101]. Developing a high-power amplifier that can efficiently amplify the pump light, along with pump-wavelength modulation techniques that enable the precise tuning of the Raman gain spectrum, is essential to overcome these limitations. Addressing these challenges can significantly enhance the performance and adaptability of the SRS light source, ultimately enabling its integration into clinical diagnostic and physiological monitoring applications. In addition, SRS can be expanded to other domains, including Raman spectroscopy, SRS microscopy, and optical communications, where it serves as a versatile multispectral light source.

## Figures and Tables

**Table 1:** The characteristics of SRS light source and target chromophores for multispectral PAI. $\lambda_{Pump}$, pump light source wavelength; $P_{Pump}$, pump light source peak power; $\lambda_{SRS}$, SRS light source wavelength; $E_{Pulse}$, SRS light source pulse energy; NIR, near-infrared; SRS, stimulated Raman scattering; PRR, pulse repetition rate; PAI, photoacoustic imaging; PB NPs, Prussian blue nanoparticles; AuNRs, gold nanorods; PE, polyethylene; PP, polypropylene; and PI, polyimide.

Spectral Region	Type	$\lambda_{Pump}$ [nm]	$P_{Pump}$ [kW]	PRR [kHz]	$\lambda_{SRS}$ [nm]	$E_{Pulse}$ [nJ]	Target	Ref.
Visible	SRS	532	-	-	532, 600	1–5	Wright–Giemsa stain	[80]
		532	8.89	5	532, 700	300	Hemoglobin, PB NPs	[81]
		532	1.875	40	532, 545, 560, 590	300–500	Hemoglobin	[82]
		532	-	800	532, 558	200	Hemoglobin	[83]
		532	-	4	532, 545, 558	100	Hemoglobin	[85]
		532	-	1000	532, 558	64–85	Hemoglobin	[87]
	Polarization- modulated SRS	532	1.5	300	532, 655	200	Hemoglobin, AuNRs	[88]
NIR	SRS	1064	13.7	7.4	1064, 1100, 1175, 1225, 1275, 1325	-	Lipid phantom	[90]
	Injection-seeded SRS	1047	10	2.5	1048, 1098, 1153, 1206, 1275	-	Drosophila larva	[91]
		1068	-	200	1192	467	White adipocytes	[92]
		1030	-	200	1168, 1202	271	PE, PI, PP	[93]
	Synchronously pumped SRS	1580, 1590, 1600	~0.264	~130	1700, 1710, 1720	400	PE, PI, PP	[94]

## Data Availability

Not applicable.
